# Membrane Separation of the Base-Catalyzed Depolymerization of Black Liquor Retentate for Low-Molecular-Mass Compound Production

**DOI:** 10.3390/membranes9080102

**Published:** 2019-08-16

**Authors:** Kena Li, Basel Al-Rudainy, Mingzhe Sun, Ola Wallberg, Christian Hulteberg, Per Tunå

**Affiliations:** 1 Department of Chemical Engineering, Lund University, P.O. Box 124, SE-221 00 Lund, Sweden; 2 Department of Chemistry, Centre for Analysis and Synthesis, Lund University, P.O. Box 124, SE-221 00 Lund, Sweden

**Keywords:** Kraft lignin, depolymerization, membrane filtration, low-molecular-mass compounds

## Abstract

One way of valorizing the lignin waste stream from the pulp and paper industries is depolymerizing it into low-molecular-mass compounds (LMMC). However, a common problem in the depolymerization of Kraft lignin is the low yields of small aromatic molecules obtained. In the present work, the combination of the repeated depolymerization of lignin and the separation of LMMC from depolymerized lignin to upgrade them into value-added chemicals was studied. In so doing, we investigated the possibility of depolymerizing black liquor retentate (BLR). The base-catalyzed depolymerization of BLR was performed using a continuous flow reactor at 170–210 °C, with a 2 min residence time. The results obtained indicate that BLR can be depolymerized effectively under the experimental conditions. Depolymerized lignin LMMC can be successfully separated by a GR95PP membrane, and thus be protected from repolymerization. Through combining membrane filtration with base-catalyzed depolymerization, more than half of the lignin could be depolymerized into LMMC. Around 46 mg/g of lignin monomers (guaiacol, vanillin, acetovanillone, and acetosyringone), which can potentially be upgraded to high-valued chemicals, were produced. On the basis of our results, we suggest use of a recycling Kraft lignin depolymerization and filtration process for maximizing the production of LMMC under mild alkaline conditions.

## 1. Introduction

Increasing interest has been directed over recent decades at the use of lignin, the most abundant natural source of aromatic polymers, as a feedstock for the production of renewable chemicals and fuels [1]. Lignin is currently treated as a by-product in many different processes, such as the Kraft process, the soda process, the sulphite process, and the second-generation bioethanol bio-refinery process. The Kraft process is the dominant chemical pulping process used worldwide in the paper and pulp industries, NaOH and Na_2_S being used in that context as chemicals for digesting lignocellullosic materials in order to obtain cellulose fibers. Around 130 million tons of Kraft pulp are generated annually [2]. During the Kraft process, more than 90% of the lignin is degraded into fractions and is dissolved in alkali solutions. It thus represents one of the major constituent of Kraft black liquor. To date, the only use of Kraft lignin in black liquor is to be burned as an internal fuel for the recovery of pulping-reagents and for power generation [3]. However, for a modern Kraft pulp mill using energy-efficient equipment, the high level of internal energy recovery obtained leads to a stream surplus that limits the capacity of the recovery boiler. The extraction of 25–50% of the lignin does not disturb milling operations, and it can provide the opportunity for increasing the milling capacity [4]. Thus, the use of lignin obtained from black liquor as a raw material for producing valuable products is potentially as significant as the use of fibers for producing pulp and paper in modern Kraft mills [5].

Two of the main approaches to extracting lignin from the Kraft black liquor are those of acid precipitation and ultrafiltration. The acid precipitation of lignin from the black liquor through use of sulfuric acid or some other acid and carbon dioxide is the most common and easiest process [6,7]. For example, the LignoBoost process has currently been a commercially available process for the production of lignin from the black liquor [8]. The main disadvantage of acid precipitation is that the use of large amounts of acidification chemicals is required. In addition, the sodium and other impurity contents of lignin during the process are very high, so more water or acid is needed for washing it than would otherwise be required [8]. Moreover, the use of sulfuric acid for acidification would disturb the balance between sodium and sulfur and increase the need to withdraw sulfates together with fly-ash from the recovery boiler fly-ash, which would seriously affect the recovery of cooking chemicals [9]. In previous research, this was solved by membrane filtration of the solution so as to separate the lignin from the pulping chemicals prior to acidification [10]. This was accomplished through the use of ultrafiltration or nanofiltration membranes for reducing the concentration of the pulping chemicals and increasing the concentration of lignin in the black liquor [10]. The black liquor that was studied contained lignin with a molecular weight of less than 10 kDa, this resulting in the authors selecting membranes in the molecular weight cut-off (MWCO) region of 1000 to 200 Da. Lignin retentions of greater than 80% have been achieved with use of maximal initial fluxes of around 200 L/m^2^h. The densest membranes had a high retention of ash, which led to the authors choosing the membrane with the highest MWCO levels for further studies. Other studies have shown that the separation of lignin into low and high molecular weight fractions is possible through use of ultrafiltration membranes [11]. The fractions obtained had lignin that differed in molecular weight but had the same main structure, as evidenced by nuclear magnetic resonance (NMR) and Fourier-transform infrared spectroscopy (FTIR) results. Further results were obtained by Padilla et al. [12], where successful fractionation of the lignin was achieved through use of a series of ultrafiltration membranes with varying MWCO (100, 30, 10, 5, and 1 kDa). The lignin obtained showed an increase in surface activity as the molecular weight increased due to the decreasing hydrophilic groups. The study also showed that the lignin of high molecular weight had a strong impact on the viscosity of the solution, which in turn could possibly affect the membrane filtration capacity. Kraft lignin was also fractionated in another study through use of sequential ultrafiltration membranes with different MWCO [13]. The different fractions contained lignin that was more homogeneous in terms of molecular weight and polydispersity than that in non-filtrated specimens. The authors have also shown that the effects on the lignin of various functional groups, differing, for example, in the quantity of the hydroxyl groups that are present, is a function of the molecular weights. A fractionation of wheat straw and Sarkanda grass lignin in a soda pulp process was performed by Allegretti et al. [14]. Their results showed a multi-step filtration process that included microfiltration and ultrafiltration to yield different lignin fractions of varying molecular weight. The high-molecular-weight fractions were proposed as being suitable as a starting material for further depolymerization of the lignin. 

In addition to the structural complexity of lignin, which represents a common challenge in the utilization of lignin, the high level of condensation that occurs during the Kraft process increases the recalcitrance of Kraft lignin [2]. To break down different lignin bonding motifs into small molecules, use of appropriate depolymerization approaches is essential. In recent years, efforts have been made to depolymerize Kraft lignin that has been extracted through acidification into low-molecular-mass compounds (LMMC). Mahmood et al. reported the depolymerization of softwood Kraft lignin at 250 °C for 45–90 min with use of NaOH as a catalyst, and this resulted in a yield of 80–90% depolymerized lignin compounds having a molecular weight ranging from 3 to 5 kDa [15]. The LignoBoost Kraft lignin was depolymerized at 290–370 °C in a near-critical water medium with use of ZrO2/K2CO3 and phenol as catalysts, and 69–87% bio-oil, 5–11% water-soluble organics, and 16–22% char were obtained after depolymerization [16]. During the lignin depolymerization process, repolymerization (or condensation) is a common and severe problem. The repolymerization mechanism can possibly be that of the electron-rich aromatic rings being easily attacked by benzyl carbocations, leading to C-C bond formation [17]. The formation of high molecular weight insoluble structures and char is the consequence of the repolymerization that occurs. In order to suppress repolymerization, we have developed a novel continuous flow reactor (CFR) to depolymerize ‘Indulin AT’ (a commericial Kraft lignin that was precipitated from the black liquor of softwood pulp) at 170–240 °C for 1–4 min of residence time by using NaOH as a catalyst [18]. We found that, under the experimental conditions, some of the Indulin AT macromolecules were depolymerized into LMMC. 

For Kraft lignin samples obtained from a membrane separation process conducted at a high pH level, the internal reagent NaOH represents a desirable catalyst for depolymerizing it to obtain LMMC. In our previous depolymerization study, most of the lignin macromolecules could not yet be depolymerized under these mild conditions. Increasing the temperature and the reaction time, or repeating depolymerization by recycling the product stream, could be effective in improving yields. At the same time, the repolymerization reactions obtained could be more severe. Using a suitable membrane to separate the LMMC from depolymerized lignin samples could be an effective option for further depolymerizing the lignin macromolecules and, at the same time, minimizing the repolymerization that occurs. To the best of our knowledge, few additional reports have been made regarding the continuous ultrafiltration and depolymerization of lignin for producing LMMC. The aim of this study was to develop a process that combined base-catalyzed continuous-flow depolymerization and membrane separation, so as to increase the yield of high-value aromatic compounds originating from the Kraft lignin. The feedstock employed in this study was black liquor retentate (BLR) obtained from a membrane filtration pilot plant. The depolymerization was performed within the temperature range of 170–210 °C, at a pressure level of 120–140 bar, and with a 2 min residence time. NaOH was the only catalyst employed in the study. The GR95PP membrane was used for the separation of LMMC after each depolymerization of the BLR samples. SEC, 2D HSQC NMR, and HPLC were used to analyze the lignin products that were obtained.

## 2. Materials and Methods

### 2.1. Materials

The BLR used in this work was obtained from a membrane filtration pilot plant situated in a pulp and paper mill in northern Sweden. The black liquor was a mixture from the softwood (70%) and hardwood (30%). The black liquor had been filtered continuously at 110 °C by a 1 kDa cut-off ceramic membrane. After membrane separation had taken place, BLR was obtained as a retentate containing 32.9% total solids, 22.4% total lignin, and 6.5% ash.

The total dry solid (TDS) content of each of the BLR samples was determined from the difference in weight before and after drying the BLR in a weighted porcelain crucible at 105 °C for 24 h. After drying, the samples were heated to 575 °C and were kept at that temperature for 3 h. The ash content of the samples was determined by weighing the residue again after it had been cooled to room temperature in a desiccator. The total amount of lignin was determined by measuring the UV absorbance at 280 nm by a spectrophotometer (UV-160, manufactured by the Shimadzu Corp., Kyoto, Japan). The absorption coefficient used was 24.6 g L^−1^ cm^−1^ [10]. The samples were diluted by a 0.5% NaOH solution. The content of polysaccharides and of Klason lignin was determined after a standard two-step acid hydrolysis of the samples, in line with NREL procedures [19]. The monomeric sugar concentration was measured using high-performance anion-exchange chromatography coupled with pulsed amperometric detection in an ICS-3000 chromatography system (Dionex Corp., Sunnyvale, USA), running at 30 °C. The sugars were separated using a CarboPac PA 1 column containing deionized water as an eluent, 200 mM of sodium hydroxide being added post-column at flow rates of 1.0 and 0.5 mL/min, respectively. The injection volume was 10 µL, and standard solutions of D-galactose, D-glucose, D-mannose, D-xylose, and L-arabinose (Fluka Chemie AG, Buchs, Swizerland) were employed. The amounts of the hemicelluloses involved were determined after anhydro-corrections of 0.9 and 0.88 for the hexoses and the pentoses respectively. Sodium hydroxide, hydrochloric acid (37%), DMSO-*d*6, ethyl acetate and all the other chemicals employed were purchased from Sigma-Aldrich Sweden AB.

### 2.2. Depolymerization of BLR

The base-catalyzed BLR depolymerization experiments were performed in a continuous flow reactor (CFR) setup. The BLR was diluted 5 times by a 2 wt % NaOH solution to obtain a solution with 5 wt. % lignin, and was filtered prior to the depolymerization experiments. The experimental setup was described in greater detail in an earlier work [18]. It is composed of an HPLC pump, a tubular CFR reactor, a filter, and a pressure control valve. A tubular preheater with a volume of 28 mL (out of which 6 mL is included in the reactor volume) was connected to the reactor (with a volume of 14 mL) beforehand to gradually preheat the sample to the required temperature. After the reaction had taken place, the sample stream was quickly condensed by a water bath that was connected to it, located just after the reactor. 

Prior to starting each depolymerization experiment, deionized water was used as the feedstock. When the system was heated up and was pressurized to operating conditions, the BLR sample was pumped continuously through the reactor at a flow rate of 10 ml/min. On the basis of the volume of interest in the preheater and the volume of the reactor, a flowrate of 10 mL/min corresponded to 2 min of residence time. In the present study, the experiments were performed at temperatures ranging from 170 to 210 °C, for 10 °C intervals, at pressures of around 130 bar. The experimental parameters and the conditions employed are summarized in Table 1. After the reactions had taken place, the 170–210 °C depolymerized BLR products were collected continuously and were stored at 4 °C for analysis and for further use. The 190 °C depolymerized BLR samples were selected for a second and a third recycling depolymerization under the same conditions to check the effects of the number of depolymerization times on LMMC production. Samples D190, 2D190, and 3D190 were obtained. 

### 2.3. Membrane Filtration and the Depolymerization Recycling

In order to separate the LMMC from the depolymerized BLR, use was made of a membrane filtration step. The type of membrane involved was an Alfa Laval membrane GR95PP, a polymeric membrane having an MWCO value of 2 kDa. The new membrane was washed at room temperature with a 4 g/L of sodium hydroxide solution prior to the diafiltration of BLR samples, in accordance with procedures described by Al-Rudainy et al. [20]. The BLR lignin samples were diafiltrated at constant temperature, transmembrane pressure and diafiltration factor values of 50 °C, 5.5 bar, and 5 g/g in a stirred pressure vessel at a calculated cross-flow velocity of 0.5 m/s [20].

**Figure 1:**
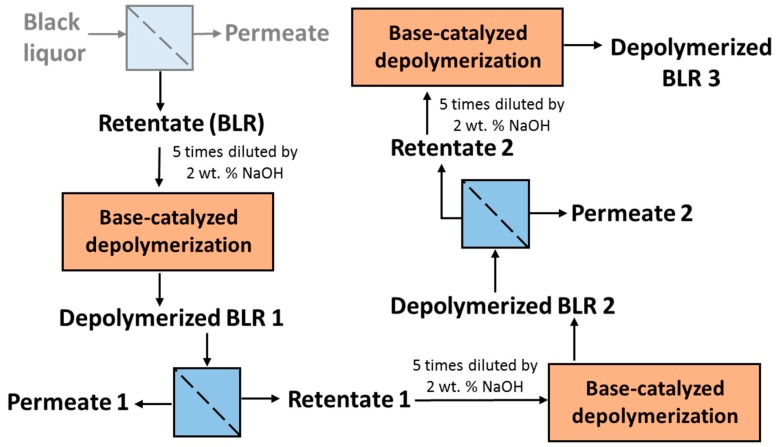
Schematic diagram of the experimental setup (Base-catalyzed depolymerization conditions: 170–210 °C, 2 min of residence time, 130 bar; membrane filtration conditions: 50 °C and 5.5 bar).

For combining depolymerization and membrane separation, the depolymerized BLR (DBLR) samples were filtered by a GR95PP membrane (2 kD MWCO) at three different temperatures, those of 170 °C, 190 °C, and 210 °C. After the membrane separation had taken place, three permeates (P170, P190, and P210) and three retentates (R170, R190, and R210) were collected for further analysis. The retentate R190 was diluted 5 times by 2 wt % of NaOH for the second round of depolymerization (with the resulting depolymerized retentate ‘2DR190’ being obtained). After the second depolymerization, another membrane filtration was performed to separate the LMMC from the 2DR190 sample, the resulting permeate (2P190) and retentate (2R190) being collected then. The retentate 2R190 was diluted 5 times again by a 2 wt % of NaOH and was depolymerized for a third time, 3DR190 being obtained. The entire process of the combined depolymerization and membrane filtration involved is shown in Figure 1.

### 2.4. Molecule Weight Distribution

The molecular weight distributions of the BLR samples used in this work were determined by means of size exclusion chromatography (SEC), making use of an *Azura* HPLC system from the firm Knauer (Berlin, Germany) containing *UVD 2.1L* and *RID 2.1L* detectors, respectively, and a *P 6.1L* pump. ClarityChrom 6.1.0 software was used to control the system. Two different *Superdx 10/300 GL* columns (*Peptide* and *200 Increase)* from GE Healthcare Bio-Sciences AB (Uppsala, Sweden) were combined to provide a high level of resolution over a large interval of molecular weights. 

The column was operated at an ambient temperature and was eluted with a 0.1 M NaOH solution as the mobile phase. All the samples were diluted by the 0.1 M NaOH eluent to a concentration of 5–10 g/L lignin, and were then passed through a 0.2 µm filter (Schleicher and Schuell, Dassel, Germany) and were prepared then for a molecular weight distribution analysis. A 50 µL amount of the diluted sample was injected and elution was performed for a period of 100 min, using an aqueous solution containing 0.1 M NaOH. For each lignin sample, two analyses were performed, the one on the *Peptide* column and the other on the *200 Increase* column. The data from the two columns was processed and then combined to provide a visualization of the molecular weight distribution of each detector signal.

### 2.5. Extraction of Lignin LMMC

Initial 10 mL amounts of BLR, depolymerized BLR, or membrane-filtrated samples were acidified by 6 M HCl to pH 1-2, 3-Ethoxy-4-hydroxybenzaldehyde (1 mg/mL, 2 mL) being added to the lignin sample prior to acidification as an internal standard. Following acidification, each sample was centrifuged to separate the supernatant and the precipitate. The precipitate was washed 3 times by acidic water, and was then freeze-dried and was stored thereafter for further analysis. The supernatant was mixed with the washing water, and was extracted with use of 10 mL ethyl acetate three times. The extract was collected in a sample tube of known weight. The solvent was then evaporated during a gentle flow of N_2_, the sample tube containing the oily LMMC product that was left then being weighed. The lignin oil (the extracted LMMC) was stored thereafter at −20 °C in a freezer until analysis was carried out. Taking into consideration the recovery obtained with use of an internal standard, the theoretical yield of the lignin oil was calculated, based on the ratio of the weight of the lignin oil to that of the initial total lignin content.

### 2.6. Lignin Aromatic Monomers Analysis

The lignin oil was dissolved in a 3 mL acetonitrile-water mixture (50:50 vol.), filtered through a 0.45 µm syringe filter prior to an HPLC analysis being performed. The HPLC analysis was carried out using an Agilent UHPLC system consisting of a degasser (G4225A, Agilent Technologies), a binary pump (G4220A, Agilent Technologies), a thermostated column compartment (G1316C, Agilent Technologies) and a diode array detector (G4212B, Agilent Technologies). An Agilent Eclipse Plus C18 column (2.1 mm × 100 mm, 1.8 µm) was used for this purpose. The flow rate was set at 0.3 mL/min. The mobile phase consisted of: A) water; and B) acetonitrile. The gradient started at 10% B and then ramped up to 30% within a period of 8 min. The amount of the acetonitrile was then increased to 90% within a period of 1 min. The gradient was then maintained at 90% acetonitrile for 3 min before being rapidly decreased during a period of 1 min to the starting composition for re-equilibration, in which it remained for 3 min. The column temperature was held constant at 45 °C. The sample volume that was injected was 5 μL. The separation was monitored with use of diode array detector (DAD) at 280 nm with use of 10 Hz as the sampling frequency. System control and data processing were performed using Agilent Chemstation software.

### 2.7. NMR Analysis

The NMR characterization carried out was performed on a Bruker Avance III HD at 500 MHz, 80 mg of lignin oil from D190 and 100 mg of freeze-dried precipitates from BLR, D190, 3D190, and 3DR190 then being dissolved in 0.5 mL of deuterated dimethyl sulfoxide-*d_6_* (DMSO-*d_6_*). The ^1^H and 2D ^1^H-^13^C HSQC NMR spectra obtained were recorded using standard pulse sequences from Bruker. The spectra were processed using MestReNova software (version 9.0).

## 3. Results and discussion

### 3.1. Base-Catalyzed Depolymerization of the BLR

Table 2 shows that the total solids, the Klason lignin, and the hemicellulose content of the BLR decreased following depolymerization. In the BLR raw material, the total solids and the Klason lignin content of the feedstock in the study were 84.1 and 39.9 g/L, respectively (Table 2). There was a total of 5.52 g/L of hemicellulose to be found in the raw material. 3 g/L of total solids was removed from the 170–190 °C depolymerized BLR. An obvious decrease in the content of Klason lignin was also found after the reaction that occurred under each of the three conditions that were present, which indicated that the BLR was effectively depolymerized under the experimental condition involved. Not only the lignin, but also all of the residual hemicellulose, was depolymerized. As shown in Table 2, the hemicellulose content of the depolymerized BLR samples at 170 °C was 2.16 g/L, then decreasing to only 0.47 g/L at 190 °C, and to still less, 0.07 g/L, at 210 °C. The concentration of xylan decreased from 3.27 g/L to 0.02 g/L after depolymerization at 210 °C.

**Figure 2:**
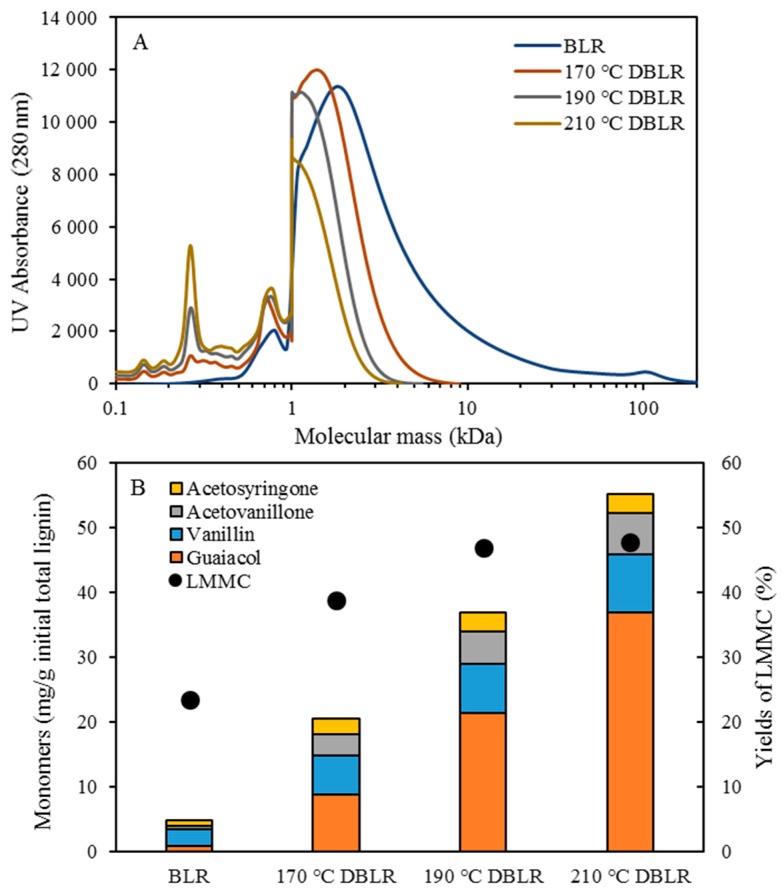
Characterization of BLR before and after depolymerization. (**A**) Molecular weight distributions of BLR and depolymerised BLR products, measured as UV absorbance at 280 nm. (**B**) The yields of lignin low-molecular mass compounds (LMMC) and the concentration of lignin monomers in LMMC before and after depolymerization. Depolymerization conditions: 5 times dilution with 2% NaOH, 170–210 °C, 120 bar, and 2 min.

The distribution of the molecular weight of the BLR raw material and of the base-catalyzed continuously flowing depolymerized BLR is shown in Figure 2A. Prior to depolymerization, the BLR sample exhibited a large amount of high molecular weight lignin, the highest molecular weights involved ranging up to levels higher than 100 kDa. Less than 20% of the lignin raw material had a molecular weight of less than 3 kDa. A clear decrease in the size of lignin molecules following depolymerization could be observed. More than 85% of the BLR was found to have depolymerized into molecules smaller than 3 kDa during the depolymerization that took place at 170 °C and 2 min of residence time. With an increase in temperature from 170 to 210 °C, the highest molecular weight of BLR shifted to a level of less than 3 kDa, indicating that a rather severe depolymerization had occurred. Distinct peaks at 200–300 and 700–800 Da following depolymerization can be observed in Figure 2A, this corresponding to the production of monomeric, dimeric and trimeric compounds, and of low-molecular-weight oligomers, respectively. The depolymerization at 210 °C gave the largest peaks of small aromatic molecules (<400 Da). Similar results have been reported previously for another technical lignin, ‘Indulin AT’. Higher depolymerization temperatures there resulted in higher yields of LMMC [18]. In the present study, the base-catalyzed depolymerization of BLR yielded better results than those for Indulin AT that we reported previously, more lignin macromolecules being depolymerized under the same conditions. 

**Figure 3:**
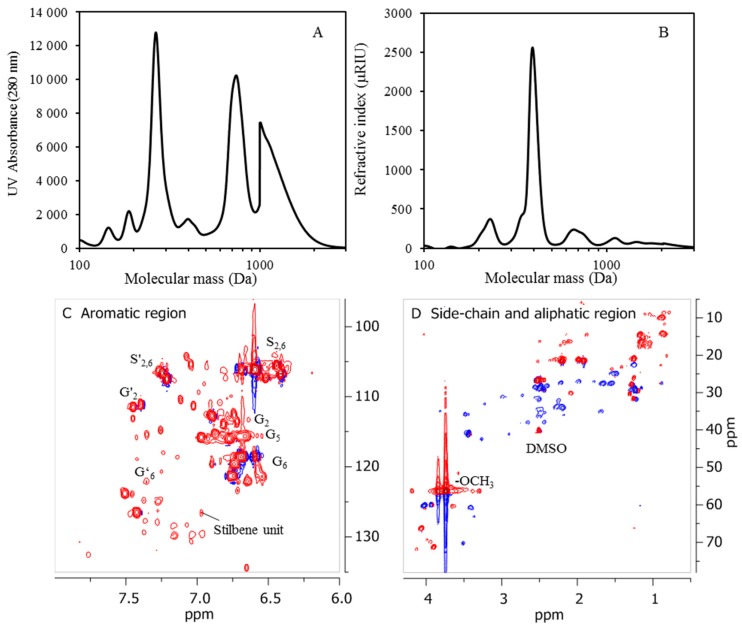
Characterization of extracted LMMC from 190 °C depolymerized BLR. (**A**,**B**) size exclusion chromatography (SEC) curves of LMMC from depolymerized BLR (measured as UV absorbance at 280 nm and refractive index). (**C**,**D**) Aromatic region, lignin side-chain, and aliphatic region of 2D HSQC NMR spectra of LMMC.

The LMMC were extracted from BLR and from depolymerized BLR. For depolymerization at 170 °C, the LMMC yields increased from 23.4% to 38.7% (of the initial lignin). When the temperature increased to 190 and 210 °C, the LMMC yields increased to 46.8% and to 47.7%, respectively (Figure 2B). The average molecular weight of LMMC was around 1000 Da (Figure 3A,B). Aromatic monomers such as guaiacol, acetovanillone, vanillin, acetosyringone, and syringaldehyde can be detected in the extracted LMMC from depolymerized BLR samples as well as from BLR raw materials. In the present study, four main monomers were selected for the monomer product comparisons and evaluations. As shown in Figure 2B, guaiacol is the main monomer product that can be produced after depolymerization, followed by vanillin, acetovanillone, and acetosyringone, in that order. Since 70% of the BLR raw material was softwood lignin, which has G-type lignin as its main structure, most of the monomers that could be detected here were G-type aromatic compounds. The content of guaiacol in BLR was only 0.825 mg/g, its increasing to 21.3 mg/g at 190 °C and to 36.8 mg/g at 210 °C. The content of vanillin, acetovanillion, and acetosyringone also showed a clear increase following depolymerization. The results regarding this show base-catalyzed depolymerization to be effective in producing LMMC from Kraft lignin. 

**Figure 4:**
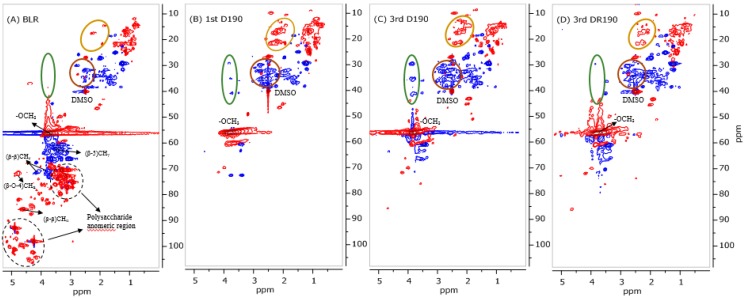
The lignin side-chain and the aliphatic region of 2D HSQC NMR spectra of the heavy fractions from (**A**) BLR, (**B**) first 190 °C depolymerized BLR (D190), (**C**) third 190 °C depolymerized BLR (3^rd^ D190), and (**D**) third 190 °C depolymerized retentate after two rounds of depolymerization and two rounds of membrane separation (3rd DR190).

Two-dimensional heteronuclear single-quantum coherence (2D HSQC) NMR experiments were conducted to determine the lignin units and the interunit linkages in BLR that were detected before and after depolymerization, respectively. The aromatic region, the lignin side-chain, and the aliphatic region of lignin oil that could be extracted from 190 °C depolymerized BLR (D190) is shown in Figure 3C,D. The lignin side-chain and the aliphatic region 2D HSQC spectra of the heavy fractions that were precipitated from both of BLR raw material and 190°C depolymerized BLR are shown in Figure 4A,B. Since the BLR sample was produced from a mixture of 30% hardwood and 70% softwood as raw material, both guaiacyl (G) and syringyl (S) units from the aromatic region could be found (δ_C_/δ_H_ 100–135/8.0–6.0 ppm) (Figure S1). Similar results were obtained for the heavy fractions from BLR samples both before and after depolymerization. The C_2_-H_2_, C_5_-H_5_, and C_6_-H_6_ correlation of G-type lignin units showed a prominent signal at δ_C_/δ_H_ 112.5/6.64, 115.7/6.68, and 119.1/6.76 ppm, respectively. The S-type lignin units and the α-oxidized S units that were related to C_2,6_-H_2,6_ are to be found at δ_C_/δ_H_ 104.0/6.62 ppm and 106.2/7.3 ppm (Figure S1). Clear C-H peaks that represent both S-type and G-type lignin that can be seen in the depolymerized lignin oil that was obtained, indicating a sharp decrease in molecular mass of the BLR lignin molecules (Figure 3C). It has been reported that a signal at around δ_C_/δ_H_ 126/7 ppm can be attributed to the C_α/β_-H_α/β_ of the stilbenes units [21,22,23,24]. In the present work, stilbenes could be observed in all of the BLR samples involved, both before and after depolymerization (δ_C_/δ_H_ 126.6/6.83–6.97 ppm) (Figure 3C and Figure S1). This shows the structure of the stilbenes to remain stable under alkaline conditions in the work reported on here. Even though β-O-4 is the bond most frequently found in lignin, most such bonds are degraded during the Kraft process [25]. Cα-Hα from β-O-4 bonds was found to be present to a slight degree at δ_C_/δ_H_ 71.6.0/4.78 ppm (Figure 4A). Because of the condensation that occurred during the Kraft process, β-β and β-5 could be clearly seen in the BLR raw material that was available. Figure 4B shows that, after depolymerization, the signals for the β-β and β-5 linkages are almost completely absent (δ_C_/δ_H_ 70–60/4–2.5 ppm). At the same time, a greater number of the –CH/–CH_3_ and of the –CH_2_ groups were observed after depolymerization had taken place, both in the case of the heavy fractions and of the lignin oil. The peaks found at δ_C_/δ_H_ 29.3/3.85, δ_C_/δ_H_ 35.4/3.83, δ_C_/δ_H_ 40.8/3.85 represent three types of methylene bridge isomers (*o-o’*, *o-p’*, and *p-p’*, respectively) [26] that appeared in the heavy lignin fraction following depolymerization (Figure 4A,B). The polysaccharide aromatic regions also disappeared after depolymerization. The results, as described here, are also in accordance with the sugar content of BLR (Table 2). 

### 3.2. Multiple Cycle Base-Catalyzed Depolymerization without Membrane Filtration

**Figure 5:**
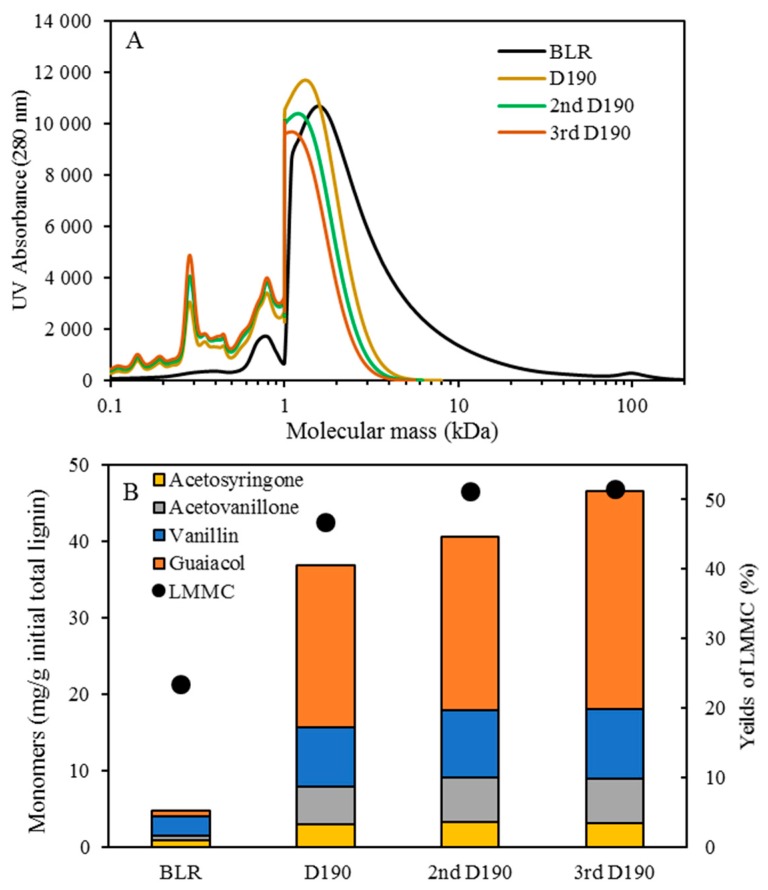
Characterization of BLR with 1-3 rounds of repeating depolymerization in 190 °C. (**A**) Molecular weight distributions of BLR and the first, second, and third depolymerized BLR products, measured as UV absorbance at 280 nm. (**B**) The yields of lignin LMMC and concentration of lignin monomers in LMMC before and after depolymerization of 1–3 rounds.

To avoid the formation of char, neither higher temperatures nor longer residence times were used during the depolymerization carried out in the present work. Instead, multiple depolymerization at 190 °C (with 2 min of residence time) was carried out so as to increase the yield of LMMC. The SEC profiles of 1–3 rounds of depolymerized BLR obtained at 190 °C are shown in Figure 5A. Repeated depolymerization gradually increased the yield of LMMC. For peaks within the range of 200–500 Da, with the lower possibly corresponding to monomers and higher to dimers, the peaks being generally higher after the second depolymerization. A further increase in the peaks within that range could be observed after the third depolymerization. Figure 4B shows the LMMC content and the monomer content after the first, second, and third depolymerization, each of them run at 190 °C. The yield of LMMC for the third depolymerization was 51.5% (0.51 g/g initial lignin) as compared with 46.8% and 51.1%, for the first and the second depolymerization, respectively, as can be seen in Figure 5B. The LMMC yields for the second and the third depolymerization, carried out at 190 °C, are also higher than the yield for the first depolymerization, carried out at 210 °C (Figure 5B). As regards the monomer content in LMMC, guaiacol is still the dominant monomer product, having increased from 21.3 mg/g of the initial lignin in the case of the first depolymerization to 22.6 and 28.5 mg/g, respectively, obtained after the second and third depolymerization. It should also to be noted that in the second and the third treatment more vanillin, acetovanillone, and acetosyringone were produced. 

The results of base-catalyzed multiple BLR depolymerization indicate that higher depolymerization yields were obtained as the number of times that depolymerization occurred increased. Although an increase in the LMMC production occurred each time, the degree of increase dropped from one depolymerization to the next. The LMMC production increased by a factor of 2 in the case of the first depolymerization; the rate of increase in LMMC of the second and third depolymerization being significantly lower than this. Such successive limitations in the increase in depolymerization that occurs can be due to the repolymerization reactions, involving the small aromatics condensing to form oligomers and polymers. The fact of this occurs could be verified on the basis of the 2D HSQC NMR spectrum (see Figure 4B,C). One can note that the methylene bridge isomers (*o-o’*, *o-p’*, and *p-p*) that peaks at δ_C_/δ_H_ 29.3/3.85, δ_C_/δ_H_ 35.4/3.83, and δ_C_/δ_H_ 40.8/3.85, respectively, are more marked in the third depolymerized BLR sample (3rd D190) than in the first depolymerized BLR (1st D190).

### 3.3. Membrane Filtration

**Figure 6:**
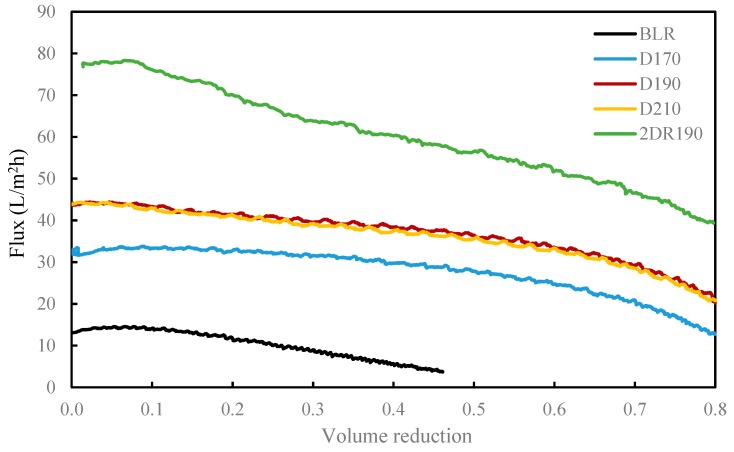
Flux during the diafiltration of 170–210 °C depolymerized BLR by GR95PP membrane. The temperature was 50 °C, the transmembrane pressure was 5.5 bar and the cross-flow was 0.5 m/s.

**Figure 7:**
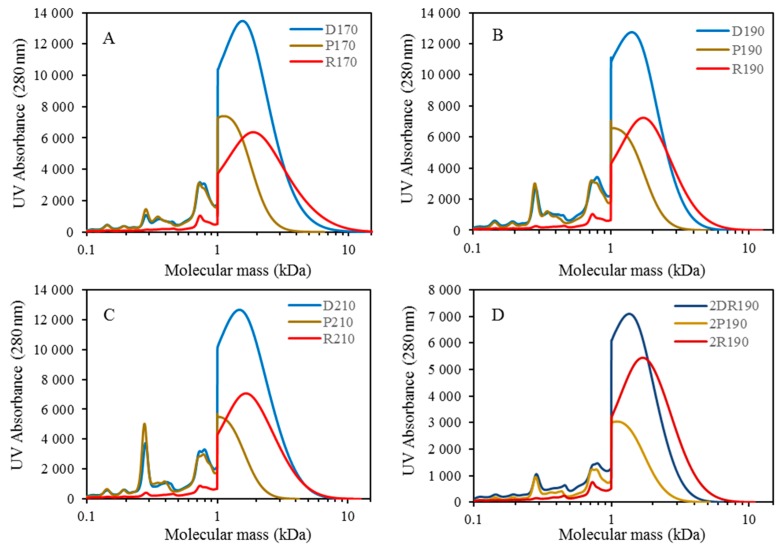
Comparison of the molecular weight distribution of untreated 170–210 °C depolymerized BLR, untreated second round 190 °C depolymerized BLR, the permeate after membrane filtration of the 4 samples, and the retentate after membrane filtration. (**A**) Untreated 170°C depolymerized BLR (D170), the permeate after membrane filtration (P170), and the retentate after membrane filtration (R170). (**B**) Untreated 190°C depolymerized BLR (D190), the permeate after membrane filtration (P190), and the retentate after membrane filtration (R190). (**C**) Untreated 210°C depolymerized BLR (D210), the permeate after membrane filtration (P210), and the retentate after membrane filtration (R210). (**D**) Untreated second round 190°C depolymerized BLR (2D190), the permeate after membrane filtration (2P190), and the retentate after membrane filtration (2DR190).

**Figure 8:**
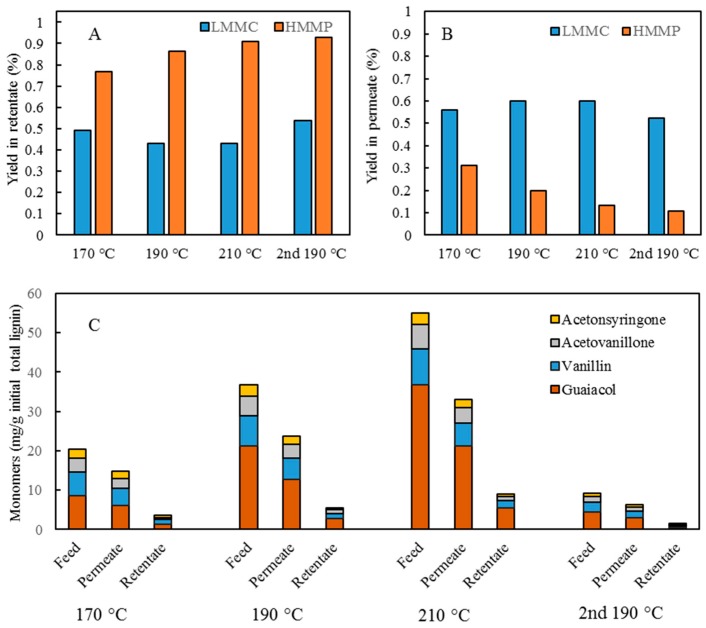
Comparison of LMMC, high molecular mass polymers (HMMP), and aromatic monomers before and after membrane filtration. (**A**) LMMC and HMMP contents in retentate after filtration. (**B**) LMMC and HMMP contents in permeate after filtration. (**C**) Monomer contents in Feed (D170, D190, D210, and 2D190) and both permeate and retentate after membrane separation.

In separating the LMMC from the depolymerized BLR, and protecting the lignin monomers, dimers, trimers, and so on, from repolymerization, additional membrane separation was performed in the present study (Figure 1). The BLR samples, depolymerized at 170–210 °C, were ultrafiltrated using the GR95PP membrane in order to separate the LMMC from one another. The initial flux showed a value of 13.1 L/m^2^ h, the value of it decreasing to 3.7 L/m^2^ h at a VR level of 46%, as can be seen in Figure 6. The flux during the filtration of a 170 °C depolymerized BLR (D170) sample increased by a factor of 2.5 (Figure 6). The flux for D190 and that for D210 were similar, the flux of both of them being higher than that of D170. The hydrolysis of polysacharides and depolymerization (at 170–210 °C) of the heavy lignin fractions contributed to an increase in the flux across the membrane (Table 2 and Figure 2A). The distribution of the molecular mass in each of the D170, D190, and D210 samples both before and after filtration is shown in Figure 6. Almost all of the high-molecular-mass polymers (HMMP) lignin was retained in the retentate (R170–210) after separation (Figure 7A–C). Figure 8 shows that 77–91% of the HMMP existed in the retentate, less than 30% being found in the permeate. The retention of HMMP increased with an increase in the depolymerization temperature. In contrast to this, the low molecular weight fractions, especially those lower than 1 kDa, were found to be readily filtered into the permeate (P170-210). Around 60% of the LMMC was recovered in the permeate (Figure 8B). Similar results for the amount of lignin monomers present before and after membrane separation are shown in Figure 8C. For the three depolymerized BLR samples, D170, D190, and D210, around 60–70% of the monomers were recovered in the permeate after separation, less than 17% of the monomers being found in the retentate. The results obtained show that the GR95PP membrane performed effectively for LMMC from the depolymerized BLR Kraft lignin.

It should be noted that around 10–20% of the monomers were lost during the membrane filtration. In our previous work, the base-catalyzed depolymerized Kraft lignin was found to be unstable at room temperature [18]. In the present study, the temperature during membrane filtration was 50 °C; it took several hours for the entire separation process to run its course. Because of this, there was a risk of repolymerization during the membrane filtration and that yields of LMMC could be higher if the filtration could be done on-stream directly after the reactor.

### 3.4. Combination of Membrane Filtration and Depolymerization

**Figure 9:**
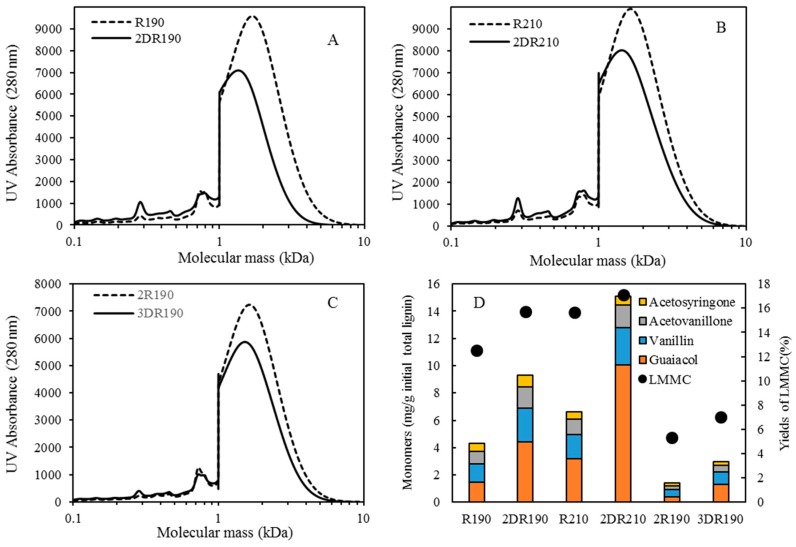
Molecular weight distributions, LMMC yields and monomer contents before and after the second round of 190–210 °C and the third round of 190 °C depolymerization (the process involving membrane separation). (**A**) SEC profiles of BLR before and after the second round of 190 °C depolymerization, measured as UV absorbance at 280 nm. (**B**) SEC profiles of BLR before and after the second round of 210 °C depolymerization. (**C**) SEC profiles of BLR before and after the third 190 °C depolymerization. (**D**) LMMC yields and the concentration of lignin monomers in LMMC from the second 190–210 °C and the third 190 °C depolymerized BLR samples.

After membrane filtration had been carried out, the retentate from the 190 and 210 °C depolymerized BLR (R190 and R210) was diluted 5 times by 2 wt % of NaOH, its being depolymerized again under the same conditions as those that were found in the first depolymerization. The SEC curves obtained are shown in Figure 9. For both of the depolymerization temperatures, the molecular weight distribution curves were found to shift toward the low molecular weight region (Figure 9A,B). Especially in the second round of depolymerization (at 190 °C), all of the molecules heavier than 5000 Da were depolymerized. A distinct peak at around 300 Da could be observed, showing the production of further monomers or dimers to have taken place after the second depolymerization had occurred. Figure 9D shows the LMMC and monomer yields obtained through the second base-catalyzed depolymerization that was carried out. The same monomer products were obtained after the first depolymerization, guaiacol and vanillin being the main monomers present at that point. The LMMC yields after the 190 and 210 °C depolymerization increased slightly from 12.5% and 15.7% to 15.7% and 17.1%, respectively. For the monomers, however, the production doubled for both 190 and 210 °C depolymerization. The yields of guaiacol increased from 1.5 and 3.2 to 4.4 and 10.1 mg/g of the initial lignin at 190 and 210 °C, respectively. 

After the second round of depolymerization, another membrane separation process was performed for the recovery of the lignin LMMC in sample 2DR190. The initial flux there was 77 L/m^2^h, which was higher than that of the separation that took place after the first depolymerization had occurred (Figure 6). The removal of depolymerized lignin after the first membrane filtration and the second depolymerization had occurred probably decreased the viscosity of the solution, resulting in the flux becoming higher. 

The SEC profile showed that the lignin of low molecular weight could clearly be separated from that of higher molecular weight (Figure 7D). However, the distribution of the molecular weight of the retentate following separation moved in the direction of the high-weight side, indicating a certain degree of repolymerization to have occurred during the membrane filtration process. Figure 7A,B also show more than 90% of the HMMP to be retained in the retentate, and 52.3% of the LMMC to be recovered in the permeate, as well as it being possible to obtain 67.1% of the monomers following membrane separation (Figure 8C).

After the second depolymerization and the membrane filtration that was associated with it, a third round of depolymerization under the same reaction conditions was performed to examine the possibility of further depolymerization. As shown in Figure 9C, a slight decrement in the molecular weight of the lignin could be observed after depolymerization (3DR190) had taken place. The monomer peaks found are present to, but to a lower extent, after the second depolymerization. Just prior to the third depolymerization, only 1.4 mg/g of the initial lignin of the monomers was left in the retentate. After that, the LMMC yields increased from 5.3% to 7.0%, and the contents of four of the monomers increased from 1.4 to 3.0 mg/g of the initial lignin (Figure 9D). The concentration of guaiacol and vanillin increased from 0.4 and 0.5 mg/g to 1.29 and 0.93 mg/g of the initial lignin, respectively. 

After the third base-catalyzed depolymerization at 190 °C and without membrane filtration, around 51% of the LMMC were obtained, in which around 46 mg/g of the initial lignin were monomers. Theoretically, without any losses occurring during membrane separation, when depolymerization and membrane filtration are combined, a total of 63% of the LMMC can be produced. Figure 4D showed that the methylene bridge isomers (*o-o’*, *o-p’*, and *p-p*) that peak at δ_C_/δ_H_ 29.3/3.85, δ_C_/δ_H_ 35.4/3.83, and δ_C_/δ_H_ 40.8/3.85, respectively, cannot be observed in lignin heavy fraction from the recycling depolymerization with membrane filtration. It illustrates that the repolymerization reaction in this process is not as severe as the recycling depolymerization without membrane filtration. In connection with this, the presence of a membrane filtration step could help to protect the LMMC that is produced, and to some extent, reduce the repolymerization reaction that occurs during the second and third depolymerization, resulting in an improvement of the depolymerization yields.

Taking into account the loss of lignin LMMC that occurs during membrane filtration, the final LMMC yield would be about 36%. For the process without membrane filtration, with the same loss of LMMC during separation from the final product, the maximum yield of LMMC that could be obtained would be 29%. Thus, the use of a membrane to separate the LMMC from the depolymerized lignin is a relatively effective way of obtaining a greater amount of aromatics of low molecular weight. Different ways of reducing repolymerization of the depolymerized lignin during membrane separation, and thus decreasing the loss in LMMC products, should be investigated further in future work.

## 4. Conclusions

It was found that the Kraft lignin BLR can be effectively depolymerized into a variety of valuable products under relatively mild conditions. About 47.7 wt % of the LMMC and 55.1 mg/g of the initial lignin of the monomers was obtained after depolymerization at 210 °C, involving a 2 min residence time. Guaiacol and vanillin are the two major monomers considered herein. Running more than one depolymerization in series (by recycling the product) resulted in higher yields of LMMC products, even though the rate of increase was lower in the second and the third rounds than in the first. Lignin macromolecules from depolymerized BLR lignin samples could be successfully separated by a GR95PP membrane for further depolymerization, and the LMMC that was recovered could be protected from repolymerization. Comparing the multiple depolymerization methods with and without membrane filtration, the former enables more LMMC to be produced than the latter. In the present work, the process involving the combination of depolymerization and membrane separation was evaluated. It was found that 63 wt % of small aromatic molecules could be obtained by running three rounds of depolymerization, and with intermediate membrane filtrations.

## Figures and Tables

**Table 1:** The experimental conditions of black liquor retentate (BLR) depolymerization.

Parameter	Range Tested
Lignin loading (wt %)	5
NaOH (wt %)	2
pH range	13–14
Temperature (°C)	170–210
Pressure (bar)	120–140
Time (min)	2

**Table 2:** Characteristics of BLR before and after depolymerization.

Concentration (g/L)	BLR	170 °C DBLR	190 °C DBLR	210 °C DBLR
**Total solids**	84.1 ± 0.07	81.9 ± 0.15	81.9 ± 1.77	80.7 ± 0.13
**Ash**	25.9 ± 0.38	25.5 ± 0.23	25.9 ± 0.38	25.7 ± 0.15
**Total lignin**	47.2 ± 0.06	-	-	-
**Klason lignin**	39.9 ± 0.28	32.3 ± 1.03	29.9 ± 0.30	27.8 ± 0.30
**Hemicelluloses**	5.52 ± 0.06	2.16 ± 0.05	0.47 ± 0.01	0.07 ± 0.00
Arabinan	1.01 ± 0.01	0.42 ± 0.01	0.08 ± 0.00	0.01 ± 0.00
Galactan	0.87 ± 0.01	0.58 ± 0.02	0.20 ± 0.00	0.03 ± 0.00
Glucan	0.12 ± 0.00	0.09 ± 0.00	0.04 ± 0.00	0.02 ± 0.00
Xylan	3.27 ± 0.04	0.99 ± 0.03	0.13 ± 0.00	0.02 ± 0.00
Mannan	0.25 ± 0.00	0.08 ± 0.00	0.02 ± 0.00	N.D.

## Supplementary Materials

The following are available online at https://www.mdpi.com/2077-0375/9/8/102/s1, Figure S1: Aromatic region of 2D HSQC NMR spectra of the heavy fractions from (A) BLR, (B) the first 190 °C depolymerized BLR (D190), (C) the third 190 °C depolymerized BLR (3rd D190), and (D) the third 190 °C depolymerized retentate after two rounds of depolymerizatio and two rounds of membrane separation (3rd DR190).
