# Supplementary material for: Membrane Separation of the Base-Catalyzed Depolymerization of Black Liquor Retentate for Low-Molecular-Mass Compound Production
Source: Membranes (Basel). 2019 Aug 16;9(8):102. doi: 10.3390/membranes9080102 (PMC6722839; doi:10.3390/membranes9080102)
Supplement: Supplementary file 1 [file membranes-09-00102-s001.pdf]

Supplementary Materials: Membrane Separation of the Base-Catalyzed Depolymerization of Black Liquor Retentate for Low-Molecular-Mass Compound Production

Kena Li [1], Basel Al-Rudainy [1], Mingzhe Sun [2], Ola Wallberg [1], Christian Hulteberg [1,*], and Per Tunå [1]

[1] Department of Chemical Engineering, Lund University, P.O. Box 124, SE-221 00 Lund, Sweden
[2] Department of Chemistry, Centre for Analysis and Synthesis, Lund University, P.O. Box 124, SE-221 00 Lund, Sweden
* Correspondence: christian.hulteberg@chemeng.lth.se; Tel.: +46 46 222 8273

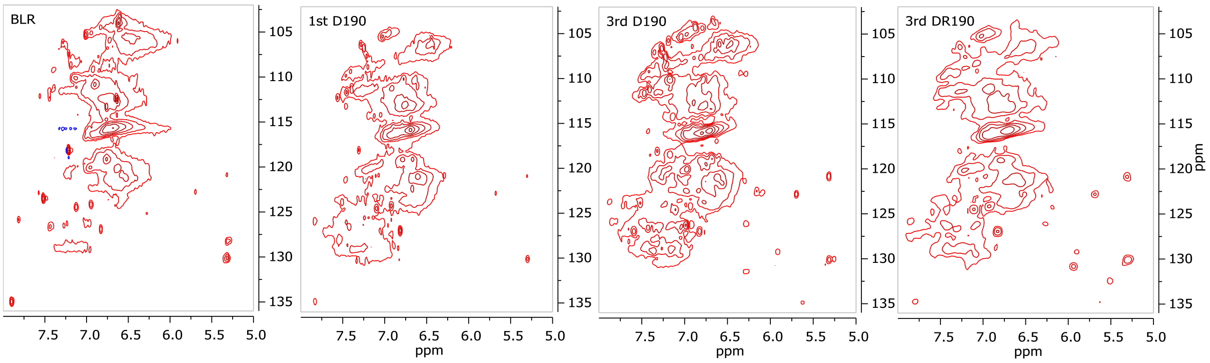

Figure S1. Aromatic region of 2D HSQC NMR spectra of the heavy fractions from (**A**) BLR, (**B**) the first 190 °C depolymerized BLR (D190), (**C**) the third 190 °C depolymerized BLR (3rd D190), and (**D**) the third 190 °C depolymerized retentate after two rounds of depolymerization and two rounds of membrane separation (3rd DR190).
